# Bipolar Disorder - New era of Treatments: High dose Levothyroxine, rTMS and genetics

**DOI:** 10.1192/j.eurpsy.2022.1040

**Published:** 2022-09-01

**Authors:** A. Zamar, T. Koutsomitros, A. Lulsegged

**Affiliations:** 1 The London Psychiatry Centre, Rtms Clinic, London, United Kingdom; 2 Maastricht University, Cognitive Neuroscience, Maastricht, The Netherlands, Greece; 3 Medical Psychotherapeutic Centre, Greek Rtms Clinic, Thessaloniki, Greece; 4 Health 121, Endocrinology And Diabetes, London, United Kingdom

**Keywords:** bipolar disorder, rTMS, High Dose Levothyroxine

## Abstract

**Introduction:**

Rapid cycling Bipolar disorder and mixed affective states are treatment resistant conditions. Standard treatments are ineffective. Homozygous polymorphism of DIO2 gene is associated 3.75-fold risk of bipolar disorder.

**Objectives:**

High Dose Levothyroxine combined with repetitive transcranial magnetic stimulation (rTMS) in Rapid cycling Bipolar disorder: Is it thyroid disease?

**Methods:**

20 RCBPD with ICD-10criteria for bipolar disorder. All were severely symptomatic. TFTs and ECGs were monitored weekly with cardiology and endocrinology backup. Genetic testing was undertaken for DiO1/DiO2 status.

**Results:**

17 were female, average age 32.4 yrs. 19/20 had Single nucleotide polymorphisms (SNP) of either DIO1, DIO2 or both. All but two patients were treated with rTMS to induce cerebral neuroplasticity. Average pre-treatment fT4 was 17.0 pmol/L , and fT3 4.5 pmol/L Average post-treatment, fT4 was 59.7 pmol/L and fT3 5.3 pmol/L. Average fT4:fT3 ratio pre-treatment was 4:1, and post-treatment was 5:1. HDL range was 200-800 mcg daily for remission. Average dose 472 mcg daily. Discontinuation rate was 0%. All patients had ECG and cardiac review One patient required a dose reduction because of minimal side effects 12 patients needed one mood stabiliser All were in remission for a minimum of 6 months.

**Conclusions:**

We speculate that BPD may be a form of cerebral hypothyroidism and that HDL helps to overcome the deficit while robust inactivating deiodinases in the periphery protect from systemic thyrotoxicosis. This is evidenced by findings of normal clinical examination and elevated rT3. rTMS exercises its well established neuroplastic effect, helping to achieve and maintain remission as an adjunct to HDL.

**Disclosure:**

The London Psychiatry Centre (TLPC) has pending patents for the combined protocol of rTMS and/or Thyroid hormones depending on the territory: - Pending US Patent application: (and/or) US20200384279 - Pending European patent appl

